# Algorithm for Improving Beneficiary Identifier Quality in Medicaid Administrative Data

**DOI:** 10.21203/rs.3.rs-9827057/v1

**Published:** 2026-05-27

**Authors:** Konstantin Kunze, Meredith C. B. Adams, Robert W. Hurley, Daniel Guth, Alina Denham, Sydney Pargman, Aniket Patil, Elaine L. Hill

**Affiliations:** University of Rochester; Wake Forest University School of Medicine; Wake Forest University School of Medicine; University of Rochester; Stony Brook University; University of Rochester; University of Rochester; University of Rochester

## Abstract

Accurately linking Medicaid beneficiaries over time and across administrative files is essential for health policy research, yet the federally assigned Chronic Conditions Warehouse (CCW) identifier and state-assigned identifier are subject to systematic data quality problems that can lead to inaccurate linkages and biased analyses. Drawing on MAX Person Summary files (1999–2015) and T-MSIS Analytic Files (2014–2022), this paper documents four key identifier problems: identifier reassignment beginning in 2020, missing identifiers, discordant identifiers, and identifier clustering. It provides a structured algorithm for correcting each. Our corrections are applied to enrollment, utilization, and mortality files, and result in a composite identifier that allows beneficiaries to be uniquely and reliably tracked over time and across files.

## Introduction

Medicaid is the largest public health insurance program in the United States, covering more than 80 million people, with enrollment and utilization data managed by the Centers for Medicare and Medicaid Services (CMS). Accurately linking beneficiaries over time and across files is essential for health policy research; however, no “gold standard” exists for this purpose, and existing methods pose problems that can lead to inaccurate linkages and biased analyses.

Two variables are used to identify Medicaid beneficiaries: the federally assigned Chronic Conditions Warehouse (CCW) identifier (BENE ID) and the state-assigned identifier (MSIS ID). As noted by Kunze et al. (2026), “administrative processes, heterogeneous state systems, and changes in federal data infrastructure can compromise identifier quality,” giving rise to linkage problems and preventing unique, one-to-one matches between federal and state identifiers. Obtaining a reliable CCW identifier requires addressing four key issues: (1) the CCW identifier bridge starting in 2020, (2) missing CCW identifiers, (3) discordant CCW identifiers, and (4) CCW identifier clusters.

Starting in 2020, CCW reassigned identifiers for a subset of beneficiaries, creating discontinuities in longitudinal tracking; a CMS-provided bridge crosswalk is needed to reconcile pre- and post-2020 identifiers. Approximately 6% of CCW identifiers are missing across the data, with California alone accounting for 28% of those missing cases. Around 3% of state identifiers are discordant, meaning they are linked to multiple CCW identifiers with conflicting demographic characteristics, suggesting incorrect assignment. Finally, 1% of CCW identifiers appear in clusters, where a single federal identifier is associated with four or more state identifiers within a given year, likely reflecting data errors rather than legitimate re-enrollment or interstate moves.

This paper provides a structured algorithm for addressing each of these four problems, offering a systematic and replicable approach to improving the reliability of Medicaid beneficiary identifiers. Addressing these shortcomings allows researchers to more accurately identify and track beneficiaries over time and across files, supporting more reliable estimates of the enrolled population and ultimately informing better health policy decisions for the Medicaid population.

## Methods

Our analysis draws on Medicaid Analytic eXtract (MAX) Person Summary files (1999–2015) and the Transformed Medicaid Statistical Information System (T-MSIS) Analytic Files (TAF; 2014–2022). We include all individuals enrolled in Medicaid or the Children’s Health Insurance Program (CHIP) for at least one day during each calendar year and exclude records flagged as having missing eligibility data. From each enrollment record we extract two key identifiers: the state identifier and the federally assigned Chronic Conditions Warehouse (CCW) identifier.

We apply a series of corrections to improve the reliability of the federal identifier. These corrections address four known problems: identifier reassignment beginning in 2020, missing federal identifiers, discordant federal identifiers, and identifier clustering.

### Federal identifier bridge.

Beginning with the 2020 TAF, CMS reassigned federal identifier values for a subset of beneficiaries, creating a discontinuity in longitudinal tracking. We applied the CMS-provided bridge crosswalk to all files from 2020 onward, replacing post-reassignment identifiers with their pre2020 equivalents when a match existed. Records without a bridge match retained their original federal identifier. This step preceded all subsequent corrections and was applied to both enrollment and claims files.

### Enrollment file corrections.

We apply three sequential corrections to the federal identifier in the MAX Person Summary and TAF Demographic and Eligibility enrollment files. The corrections proceed in a fixed order: recovery of missing identifiers, then exclusion of discordant identifiers, then exclusion of clustered identifiers. This ordering matters because each step depends on the output of the previous one. A recovered identifier, for example, may subsequently be flagged as discordant or clustered and excluded. At each step we retain indicator variables on the enrollment record that we carry forward to utilization files.

### Recovery of missing federal identifiers.

Many Medicaid beneficiaries lack a federal identifier. However, some of these individuals appear in other enrollment years with a valid federal identifier. We exploited this longitudinal variation by constructing a crosswalk from all MAX (1999–2015) and TAF (2014–2022) enrollment records. For each state identifier and state combination that mapped to exactly one non-clustered federal identifier across the full panel, we assigned that federal identifier to records in which it was missing. We retained the original federal identifier when present and substituted the crosswalk value otherwise.

### Discordant federal identifiers.

In some cases, the same state identifier within a state has been linked to multiple distinct federal identifiers across years, with substantial differences in associated demographic characteristics (sex, race/ethnicity, date of birth), suggesting that the federal identifier was assigned incorrectly. We identified these discordant cases by linking state identifiers across time and comparing the associated federal identifiers and demographics. Records flagged as discordant were marked with an indicator variable and their federal identifier was treated as unreliable and set to missing.

### Clustered federal identifiers.

A single federal identifier can be incorrectly shared across multiple unrelated individuals. We define a cluster as a federal identifier linked to four or more distinct state identifiers within a single calendar year. The cluster determination is year-specific because the set of affected identifiers changes over time. Records with a clustered federal identifier were flagged and their federal identifier set to missing.

### Composite identifier construction.

After applying all corrections, we construct a composite identifier. When a corrected federal identifier is available, the composite identifier takes that value; otherwise, it is set to a concatenation of the state identifier and state (MAX) or submitting state (TAF) code.

### Utilization file corrections.

We extend the identifier corrections to claims files (inpatient, other therapy, long-term care, and prescription drug). For claims from 2020 onward, we first apply the federal identifier bridge using the same procedure described above. We then merge corrected enrollment records to the claims files using the state identifier and state (MAX) or submitting state (TAF) code, recovering federal identifiers where they are missing on the enrollment record but present on the corresponding claim. After merging, we apply the discordant and cluster flags carried from the enrollment file: when either flag is present, the federal identifier on the utilization record is set to missing. We then construct the composite identifier on the merged file using the same logic as the enrollment files.

### National Death Index file corrections.

We link enrollment records to National Death Index (NDI) files using the same identifier corrections described above. Because death records may be linked through either the federal or state identifier but not necessarily both, we perform three sequential merges: first on both the federal identifier and the state-level composite identifier jointly, then on the federal identifier alone, and then on the state-level composite identifier alone. For files from 2020 onward, the federal identifier bridge is applied to the enrollment records prior to the NDI merge, though it is not applied to the NDI records themselves, as the NDI identifiers predate the 2020 reassignment. For further detail on the NDI linkage and data quality, see Kunze et al. (2025).

## Results

### Magnitude of federal identifier quality problems.

Four systematic problems were identified in the federally assigned Chronic Conditions Warehouse (CCW) identifier across the combined panel:

1. *Identifier reassignment*. Beginning with TAF 2020, CMS reassigned federal identifier values for an unreported subset of beneficiaries. Reconciliation across the 2020 discontinuity required application of the CMS-provided bridge crosswalk to all post-2020 files.
2. *Missing identifiers*. Approximately 6% of federal identifier values were missing across the combined MAX and TAF panel. State-level concentration was substantial: California alone contributed approximately 28% of all missing-identifier cases. State-by-state breakdowns beyond California are not presented in the current manuscript.
3. *Discordant identifiers*. Approximately 3% of state identifiers within a state were linked to multiple distinct federal identifiers with conflicting associated demographic characteristics (sex, race/ethnicity, or date of birth) across enrollment years, indicating likely misassignment. The threshold for “substantial” demographic differences is not operationalized in the current draft.
4. *Clustered identifiers*. Approximately 1% of federal identifiers were associated with four or more distinct state identifiers within a single calendar year, a pattern interpreted as a single federal identifier being incorrectly shared across multiple unrelated beneficiaries rather than as legitimate interstate movement or re-enrollment. Cluster status was determined on a year-by-year basis because the set of clustered identifiers changes over time.

### Algorithm output.

After sequential application of the four corrections (bridge crosswalk → recovery of missing identifiers from longitudinal panel structure → exclusion of discordant identifiers → exclusion of clustered identifiers), each enrollment record received: (a) a corrected federal identifier when one could be validly recovered or retained, (b) indicator variables flagging missing, discordant, and clustered status at each step, and (c) a composite identifier taking the value of the corrected federal identifier when available, or otherwise a concatenation of the state identifier and state code. The composite identifier was constructed on enrollment, utilization (inpatient, other therapy, long-term care, prescription drug), and mortality (NDI) files using identical logic, supporting unique, longitudinal beneficiary tracking across files.

### National Death Index linkage.

Death records were linked to enrollment records through three sequential merges: jointly on federal and state-level composite identifier; on federal identifier alone; and on state-level composite identifier alone. The federal identifier bridge was applied to enrollment records but not to NDI records, because NDI identifiers predate the 2020 reassignment. Further detail on the linkage and its data quality is reported in Kunze et al. (2025).

## Discussion

This paper introduces a structured, deterministic algorithm for resolving four systematic quality problems in the federal beneficiary identifier used across Medicaid administrative data: post-2020 identifier reassignment, missing identifier values, discordant identifiers linked to conflicting demographic profiles, and clustered identifiers shared across multiple unrelated beneficiaries. Applied uniformly to MAX Person Summary files (1999–2015), TAF Demographic and Eligibility files (2014–2022), and linked utilization and National Death Index files, the algorithm produces a composite identifier that supports unique, longitudinal beneficiary tracking across files and over time. The corrections address problems that are individually small in percentage terms but substantial in absolute terms when applied to a national program covering more than 80 million beneficiaries.

## Contextualization Within the Literature

Identifier instability in claims-based administrative data is a long-standing concern. Prior work has documented analogous problems in Medicare enrollment identifiers and in cross-state linkage of Medicaid beneficiaries (OIG 2020, Leonard 2017, Jones 2023, RESDAC). The broader record-linkage literature has converged on two dominant strategies: probabilistic linkage, which constructs match probabilities from demographic and identifying attributes, and deterministic linkage, which relies on rule-based application of administrative keys (Sayers 2015, Tromp 2011). The present algorithm sits squarely within the deterministic tradition but extends it in three respects: it exploits the longitudinal panel structure of MAX and TAF to recover missing identifiers from adjacent enrollment years; it applies year-specific exclusion rules to clustered identifiers; and it propagates correction flags from enrollment records into utilization and mortality files, preserving consistency across the full analytic stack.

The methodological niche is distinct. Probabilistic linkage operates on demographic similarity and is most useful when administrative identifiers are absent or unreliable across data systems. The algorithm described here instead operates on the existing federal-state identifier infrastructure of MAX and TAF, leaving the Medicaid research workflow intact while improving the reliability of the keys that workflow depends on. To our knowledge, no prior published method has addressed all four documented Medicaid identifier problems in a single, replicable pipeline applied uniformly across enrollment, claims, and mortality files (Schpero 2025).

## Strengths of the Approach

Several strengths are grounded in the design of the algorithm and its application. First, the algorithm is fully deterministic and transparent. Every correction follows an explicit rule, and every excluded or recovered record receives an indicator variable that travels with the record into downstream analyses. Researchers can therefore audit, reverse, or override any correction without re-running the pipeline. Second, the longitudinal recovery step leverages information across a 24-year panel, allowing the algorithm to assign federal identifiers to enrollment records in years when those identifiers are missing but the same state identifier maps unambiguously to a non-clustered federal identifier in other years. At an approximately 6% missingness rate, this step has nontrivial scale effects. Third, the algorithm is applied uniformly across enrollment, utilization, and NDI files, eliminating the common problem of identifier drift across linked files within the same study. Fourth, the composite identifier construction preserves all records. When no valid federal identifier can be recovered, the algorithm defaults to a state-level composite key rather than dropping the record, reducing a known source of selection bias in administrative-data research. Fifth, the algorithm explicitly handles the 2020 CMS bridge crosswalk discontinuity, which is a recent and under documented threat to longitudinal validity for any study spanning the pre- and post-2020 TAF transition.

## Figures and Tables

**Table 1:** Variables in Medicare & Medicaid Files

Description	Variable Name	File Availability
Unique CCW identifier for a beneficiary	BENE_ID	1, 2, 3
Unique state-assigned identifier for a beneficiary	MSIS_ID	1, 2, 3
Submitting State Alpha Abbreviation	STATE_CD	1, 2, 3
Submitting State Code	SUBMTG_STATE_CD	2
Indicator of Missing Eligibility Record	MSNG_ELG_DATA	2
Indicator of Missing Eligibility Record	MISG_ELGBLTY_DATA_IND	1

Notes: This table shows variables that have been used in the analysis. 1: Medicaid Analytic Extract Personal Summary File (1999–2015). 2: Medicaid T-MSIS Analytic Demographic and Eligibility File (2014–2020). 3: Medicaid National Death Index File (1999–2020).
